# Adherence to daily growth hormone therapy in Japan: real-world data from the Melon Nikki^TM^ app and device

**DOI:** 10.1210/jendso/bvag113

**Published:** 2026-05-15

**Authors:** Akari Nakamura-Utsunomiya, Taisuke Ono, Osamu Arashin

**Affiliations:** Department of Pediatrics, Hiroshima North Medical Center, Asa Citizens Hospital, Hiroshima 731-0293, Japan; Department of Medical Genetics, Biomedical and Health Sciences, Hiroshima University Graduate School, Hiroshima 734-8551, Japan; Department of Pediatrics, Hiroshima University Hospital, Hiroshima 734-8551, Japan; Department of Pediatrics, Hiroshima North Medical Center, Asa Citizens Hospital, Hiroshima 731-0293, Japan

**Keywords:** growth hormone, adherence, electronic devices, smartphone application

## Abstract

**Context:**

Since 2020, a smartphone app (Melon Nikki^TM^) that is used in conjunction with the electronic drug delivery device (GROWJECTOR^TM^ L) has been available in Japan. Real-world data generated through this system enable evaluation of treatment adherence among pediatric patients receiving GH therapy.

**Objectives:**

To assess adherence to home-based GH self-injection using a smartphone application linked to an electronic injection device and identify factors associated with adherence.

**Design:**

Observational study

**Setting:**

Real-world data were collected and analyzed, including home injection patterns, GH dosage, and changes in height and body mass index over the first year of therapy. Factors significantly associated with adherence outcomes were evaluated using statistical analyses.

**Patients:**

We enrolled a cohort of Japanese pediatric patients with various indications for GH therapy who used an electronic injection device connected to the Melon Nikki^TM^ application.

**Results:**

Injection adherence declined after the second month in patients with GH deficiency and after the fourth month in patients born small for gestational age, particularly among those with variable injection times. Multivariate analysis showed that cumulative app usage up to 24 weeks is an independent factor that significantly contributes to an increase in the cumulative injection rate. Use of the application was associated with better adherence during initial 8 weeks.

**Conclusion:**

Use of a smartphone application linked to an electronic injection device may support adherence maintenance during the early phase of GH therapy. These findings provide clinically relevant insights that may inform patient guidance and adherence-focused interventions.

Growth hormone (GH) therapy has been a cornerstone treatment for children with short stature in Japan for more than 60 years [1]. Initially, GH was derived from the human pituitary glands, supplied as single-dose preparations, and administered at home by parents or other family members after reconstitution of ampules for subcutaneous injection [1, 2]. With advances in recombinant gene technology, recombinant human GH preparations were introduced in the 1980s, accompanied by the development of devices that simplified subcutaneous administration [3]. During the 2000s, further technological progress led to the introduction of motorized injection devices. In parallel with these technical advancements, the range of indications for GH therapy has expanded substantially. In addition to growth hormone deficiency (GHD), approved indications in Japan now include Turner syndrome, achondroplasia, chronic renal failure–associated short stature, small for gestational age (SGA) stature, and short stature homeobox (SHOX) disorder, which was added in 2023 [1]. Alongside the introduction of long-acting weekly GH formulations, the diversity of available GH preparations and delivery devices continues to increase [4, 5].

Despite these advances, treatment adherence remains a critical determinant of GH therapy effectiveness [6, 7]. GH is typically self-administered at home through subcutaneous injection by guardians or by patients themselves. Increased patient and family engagement and autonomy in injection management have been associated with improved therapeutic outcomes. Factors reported to influence adherence negatively include prolonged treatment duration, suboptimal growth response, injection-related pain, anxiety surrounding the injection procedure, and insufficient communication with health care professionals [6]. In contrast, survey-based studies have shown that patients who report higher satisfaction with GH injections tend to maintain better adherence to therapy [7].

Recently, a medication management smartphone application for GH therapy has been developed to support improved treatment outcomes. When used in conjunction with GH preparations, the application not only enables electronic recording of growth curves but also includes reminder alarms for injection timing, child-friendly avatar-based features to enhance engagement during treatment, and messaging functions that facilitate communication among patients, family members, and health care professionals at a distance [8]. Therefore, app-linked medical management has been suggested to have clinical utility [8]. In Japan, electronic injection devices have been in use since 2020, and the smartphone applications linked to them have since become widely available. Previous studies have reported that integrating a smartphone application influenced injection adherence in a cohort of 57 patients in Japan; however, no statistically significant results from large-scale studies have been reported [8].

In this study, we aimed to investigate the real-world status of home-based GH therapy and current adherence patterns. We further conducted statistical analyses to identify factors influencing GH treatment effectiveness and adherence using data from 1858 registered accounts collected over a 3-year period following service initiation in 2020.

## Materials and methods

### Design and setting

This retrospective observational study included Japanese pediatric patients with short stature who were receiving GH therapy with GROWJECT™ (a recombinant human GH preparation; JCR Pharmaceuticals Co., Ltd., Hyogo, Japan) in routine clinical practice and had downloaded and registered for a smartphone application linked to electronic injection devices dedicated to GROWJECT™. Injection data recorded by the device, together with data collected through the application, accumulated between October 20, 2020 and October 31, 2023, were used for analysis.

This study included data from 1858 patients and guardian accounts selected from 2211 total registrations to the smartphone application between October 23, 2020 (application released), and October 31, 2023.

### Electronic injection devices and smartphone application

**Figure 1:**
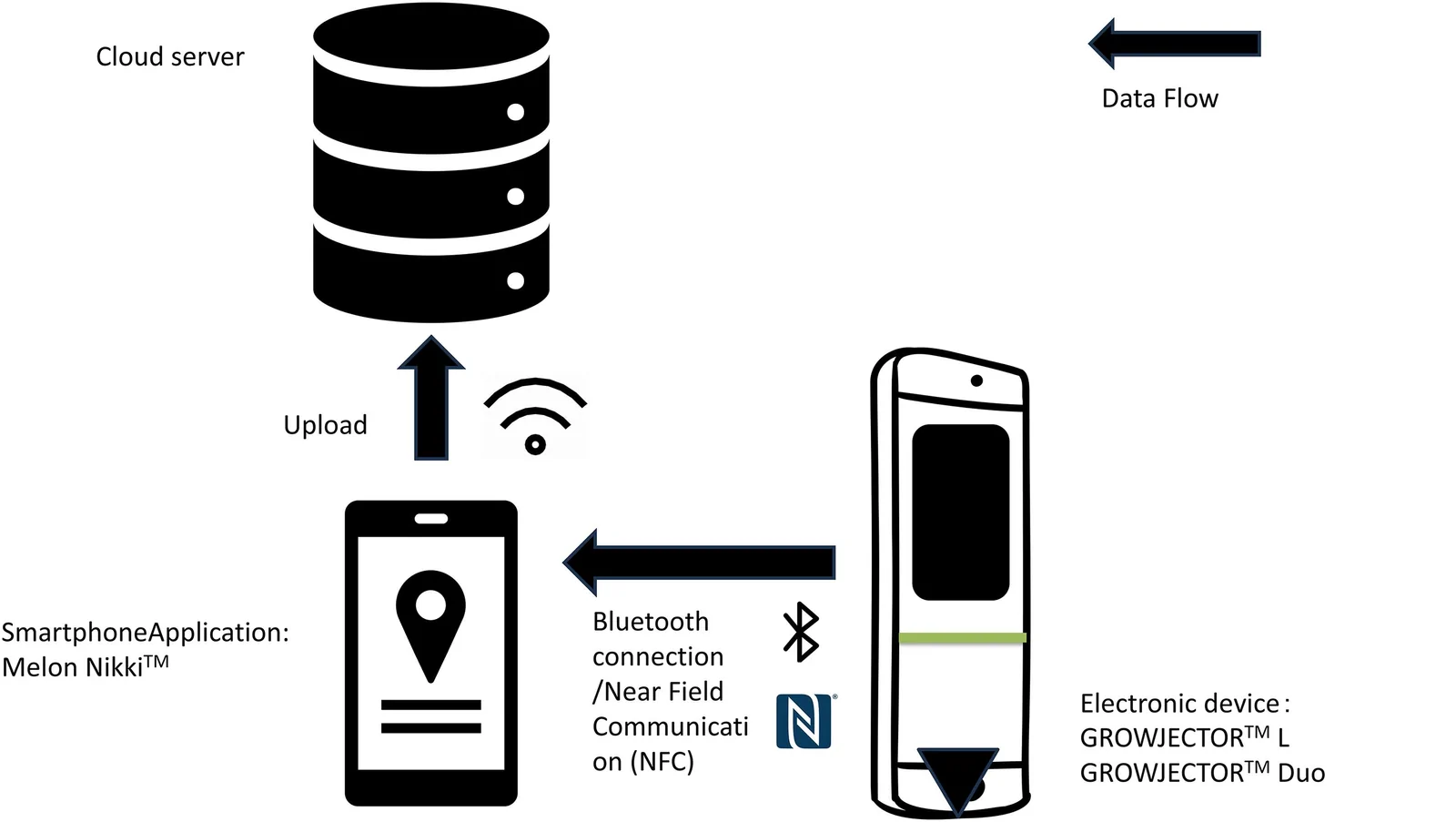
Data flow from the electronic injection device to the smartphone application and cloud server.

The electronic injection devices used in this study were GROWJECTOR^TM^L and GROWJECTOR^TM^Duo, dedicated devices for GROWJECT^TM^, linked to the smartphone application Melon Nikki™ (JCR Pharmaceuticals Co., Ltd., Hyogo, Japan). The devices store records of up to 400 injections, and these data are transferred to the application at the time of application registration and at each subsequent synchronization with a smartphone and are then stored on a cloud server (Fig. 1).

This system enables users to review detailed injection records, including the date, time, and dose of each injection. The application also records patient information such as date of birth and sex and allows entry of height, weight, and injection site. In addition, it automatically generates growth curves, provides reminder notifications for scheduled injections, and includes a message function to support remote communication. In particular, the application includes a reward-based system in which points accrue with each injection and upon achievement of injection milestones and can be redeemed for avatar items of the user's choice, thereby supporting continued treatment.

During the study period, the application was updated to allow entry of additional data fields, including treatment indication and the number of injections scheduled per week. This made it possible to perform indication-specific analyses and to more accurately assess treatment adherence.

### Analysis populations

The study population comprised the Full Analysis Set (FAS) and three analytical subsets: the Newly Treated Set (NTS), the Continuing Treatment Set (CTS), and the Extended-Data Set (EDS). The FAS included all eligible patient accounts registered for the smartphone application. Because the actual date of treatment initiation was not available, the NTS and CTS were operationally defined on the basis of the first injection record available in the device. The NTS included patients who were registered within 30 days of the first injection record and whose height standard deviation score (SDS) was below −2.0 at that time. The CTS included patients who were registered 31 days or more after the first injection record. The EDS included patients in the NTS for whom additional data on treatment indication and the scheduled number of injections per week were available.

### Study endpoints

The study evaluated three endpoints. First, growth outcomes after treatment initiation were assessed in the NTS and EDS using changes in height SDS and body mass index (BMI) SDS. Second, injection patterns in home-based GH therapy were evaluated in the FAS, including injection timing and injection site. Third, adherence-related outcomes were evaluated. In the EDS, changes in adherence rate after treatment initiation were evaluated. Adherence rate was defined as actual injection frequency divided by the number of planned injections. Factors associated with adherence rate were also explored in the EDS. In the CTS, injection frequency before and after application registration was compared. In the FAS, associations of injection timing and smartphone application engagement with injection frequency were evaluated.

### Ethics

This study was approved by the Ethics Committee of Hiroshima North Medical Center, Asa Citizens Hospital. At the time of application registration, patients or their guardians provided consent to the use of de-identified data for research purposes. Access to the data was granted by JCR Pharmaceuticals Co., Ltd. following the execution of a clinical research agreement. Only anonymized user data, from which all personal identifiers had been removed before analysis, were made available to the investigators.

### Statistical analysis

Continuous variables were summarized using means, standard deviations (SDs), and 95% confidence intervals (CIs). Categorical variables were summarized using numbers and percentages. Nonparametric statistical methods, including Mann–Whitney *U* test, Spearman's correlation coefficient, and Wilcoxon signed-rank test, were used as the primary analytical approach. For growth outcomes, linear mixed-effects models were applied to address data sparsity resulting from optional data entry by patients.

Factors associated with adherence were explored using multivariable linear regression in patients in the EDS whose injection records were available for at least 24 weeks. Because of the limited sample size, univariable linear regression was first used to screen candidate independent variables, with a threshold of *P* < .25. Given the exploratory nature of this study and absence of prespecified hypotheses, no adjustments were made for multiplicity of statistical testing.

All statistical analyses were performed using SPSS version 28.0 (IBM Corporation, Armonk, NY, USA) and R version 4.5.0 (R Foundation for Statistical Computing, Vienna, Austria). These statistical analyses were conducted by Kondo Inc., and the results were provided to the investigators.

## Results

**Figure 2:**
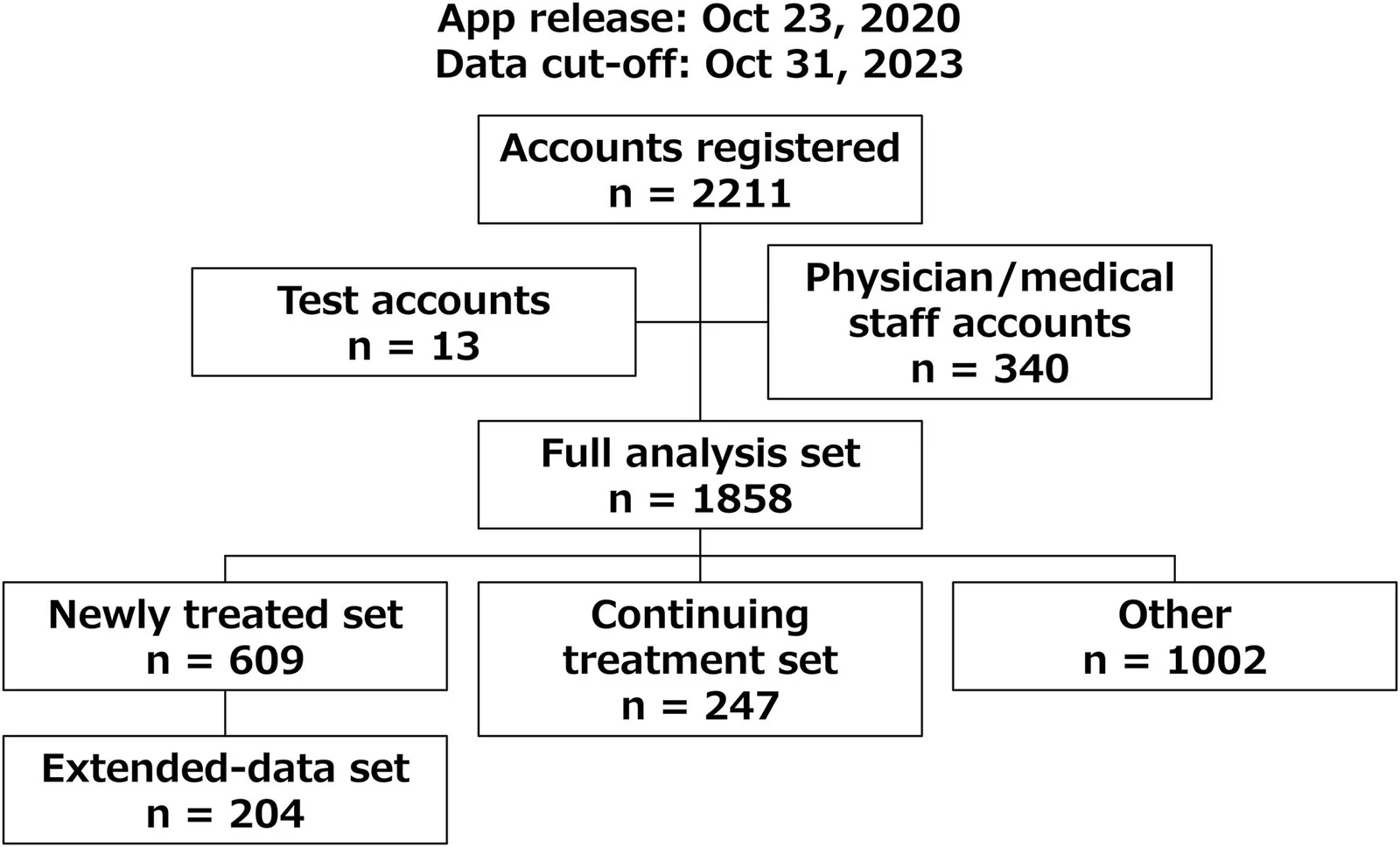
Study flow diagram.

In total, 2211 accounts were registered for the smartphone application between October 23, 2020, when the application was released, and October 31, 2023. After excluding healthcare professional accounts and test accounts, 1858 patient accounts were included in the FAS. Among these, 609 accounts met the criteria for inclusion in the NTS, as described in the Analysis populations section of Materials and Methods. Of these, additional data on treatment indication and number of scheduled injections per week were available for 204 accounts, which were included in the EDS (Fig. 2). The EDS comprised 84 patients with GHD, 23 patients with Turner syndrome or SHOX disorder, and 97 patients with SGA (Fig. 2).

### Patient characteristics

**Figure 3:**
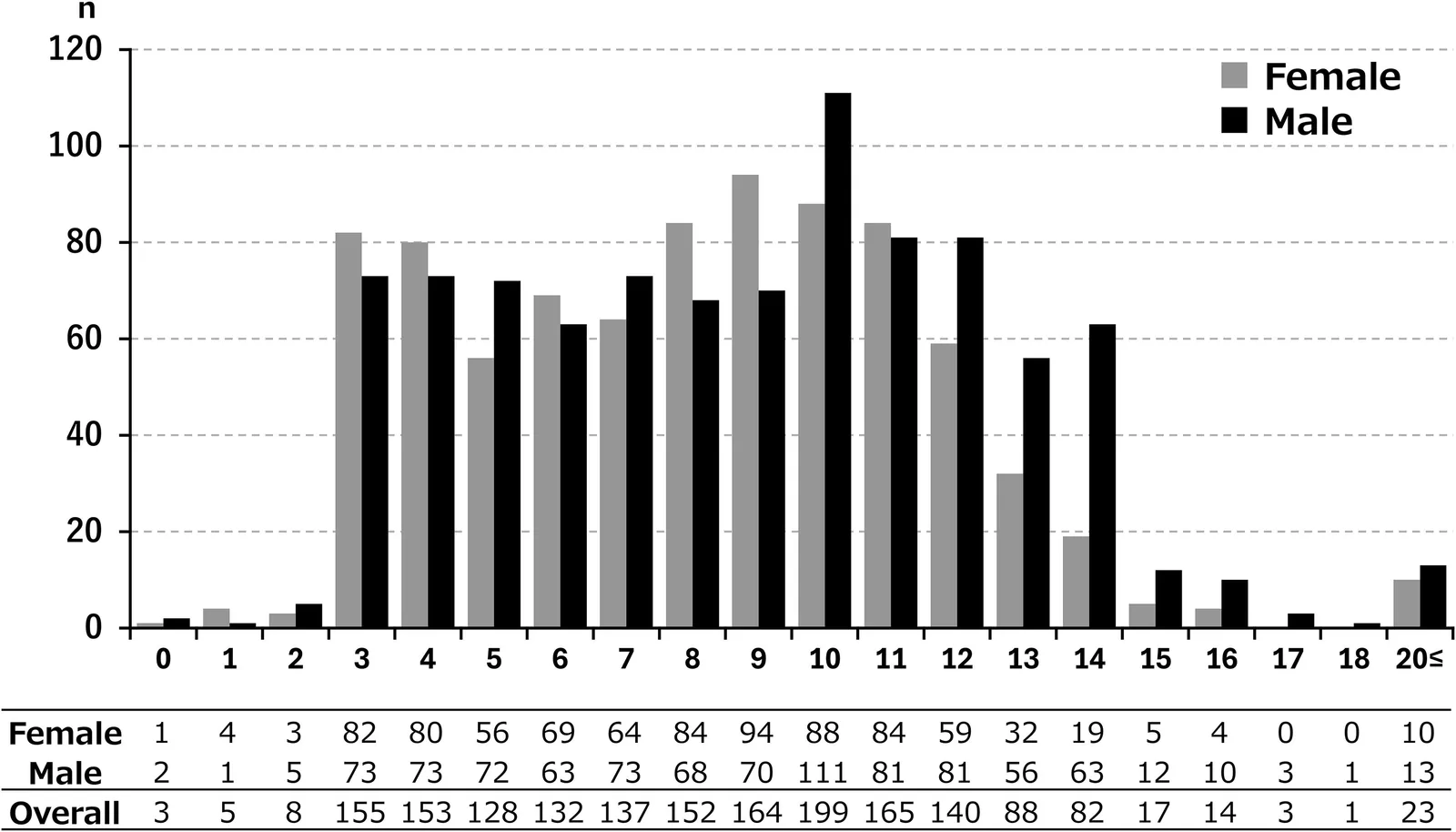
Age and sex distribution of included patients.

The number of participants by sex and age group is shown in Fig. 3. For both male and female patients, the age distribution was centered between 3 and 14 years. Female patients were more concentrated under 10 years of age, whereas male patients were more common among those aged 10 years and older.

### Changes in height SDS and BMI SDS after treatment initiation

**Figure 4:**
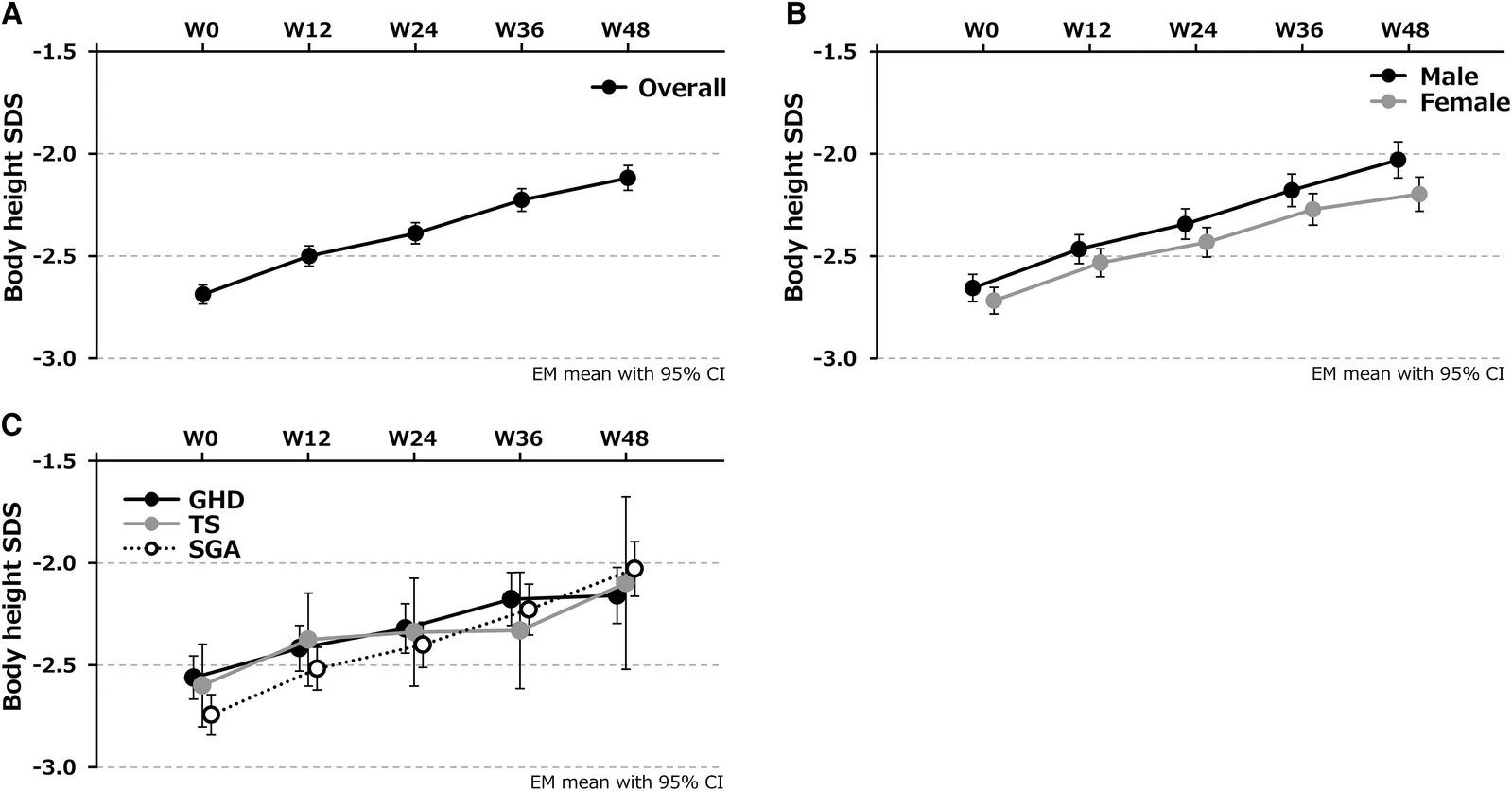
Changes in height standard deviation score (SDS) after initiation of GH therapy. (A) All patients. (B) Male and female patients. (C) Stratified by GH therapy indication.

Over 1 year of treatment, the overall NTS population demonstrated an estimated mean (95%CI) increase of 0.57 (0.51-0.63) SDS in height (Fig. 4A). When stratified by sex, boys showed a 0.63 (0.55-0.71) SDS increase in height over 1 year, whereas girls showed a 0.52 (0.45-0.60) SDS increase (Fig. 4B). Analysis by treatment indication in the EDS showed that patients with GHD experienced an estimated mean height increase of approximately 0.4 SDS after 1 year of therapy. Patients with SGA demonstrated a height increase of approximately 0.7 SDS (Fig. 4C). When stratified according to GH dosage increase at 6 months after treatment initiation in patients with SGA, height gain at 1 year was comparable between increase and non-increase groups. Patients with Turner syndrome or SHOX disorder demonstrated a height increase of approximately 0.5 SDS, at 1 year.

**Figure 5:**
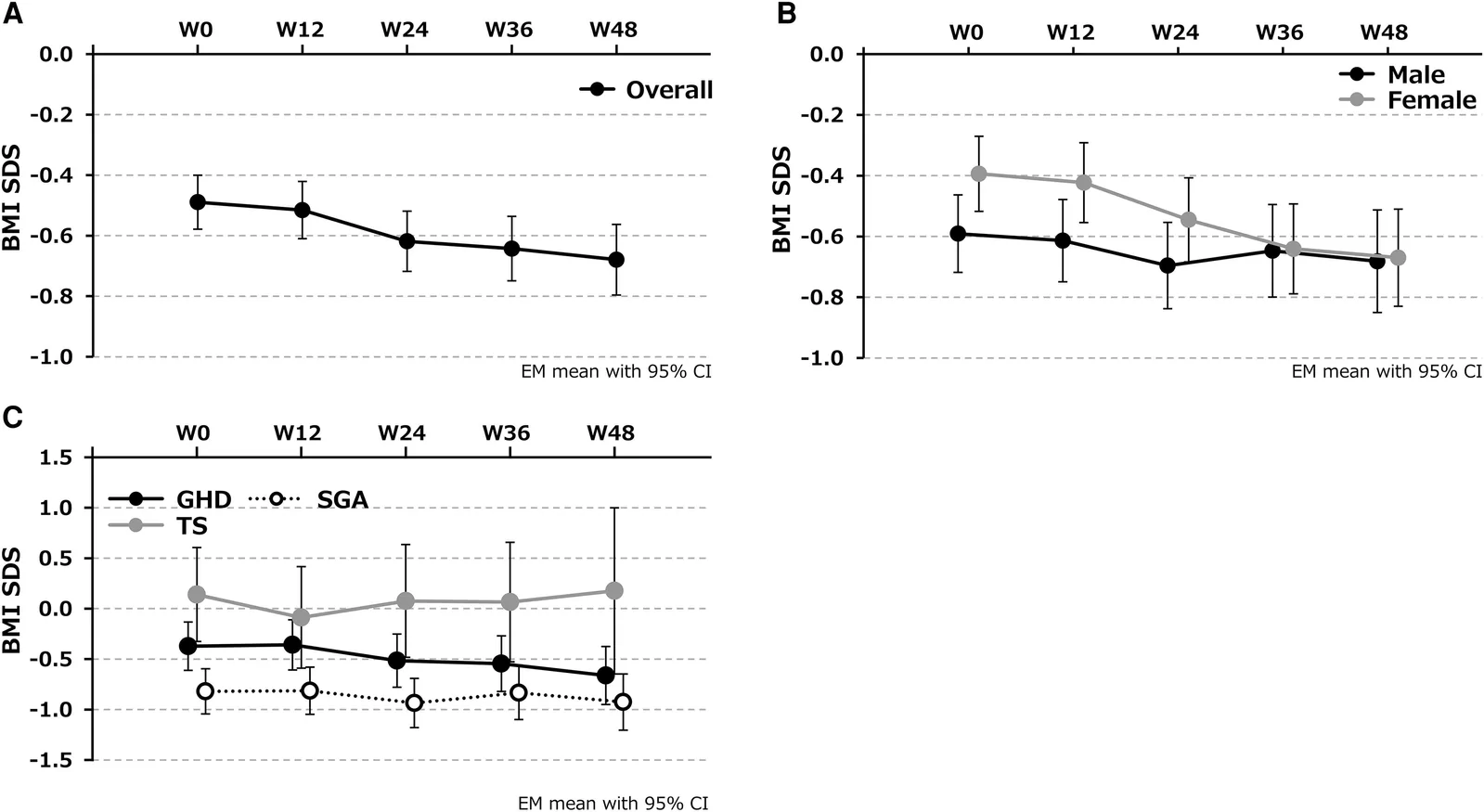
Changes in body mass index (BMI) SDS after initiation of GH therapy. (A) All patients. (B) Male and female patients. (C) Patients stratified by GH therapy indication.

Regarding BMI changes, the overall NTS population demonstrated an estimated mean (95%CI) decrease of 0.09(−0.06 to 0.25) SDS over 1 year; however, this change was not statistically significant, and BMI values remained relatively stable. In contrast, girls exhibited a significant decrease of 0.28 (0.13-0.42) SDS over 1 year, with a statistically significant decline observed from 6 months after treatment initiation (Fig. 5A). When analyzed by treatment indication in the EDS, patients with GHD demonstrated a mean decrease in BMI of 0.29 SDS over 1 year. In patients with SGA, BMI remained stable over the 1-year period, regardless of dose escalation. In patients with Turner syndrome or SHOX disorder, no clear trend was identified (Fig. 5C).

### Average injection time and injection site

**Figure 6:**
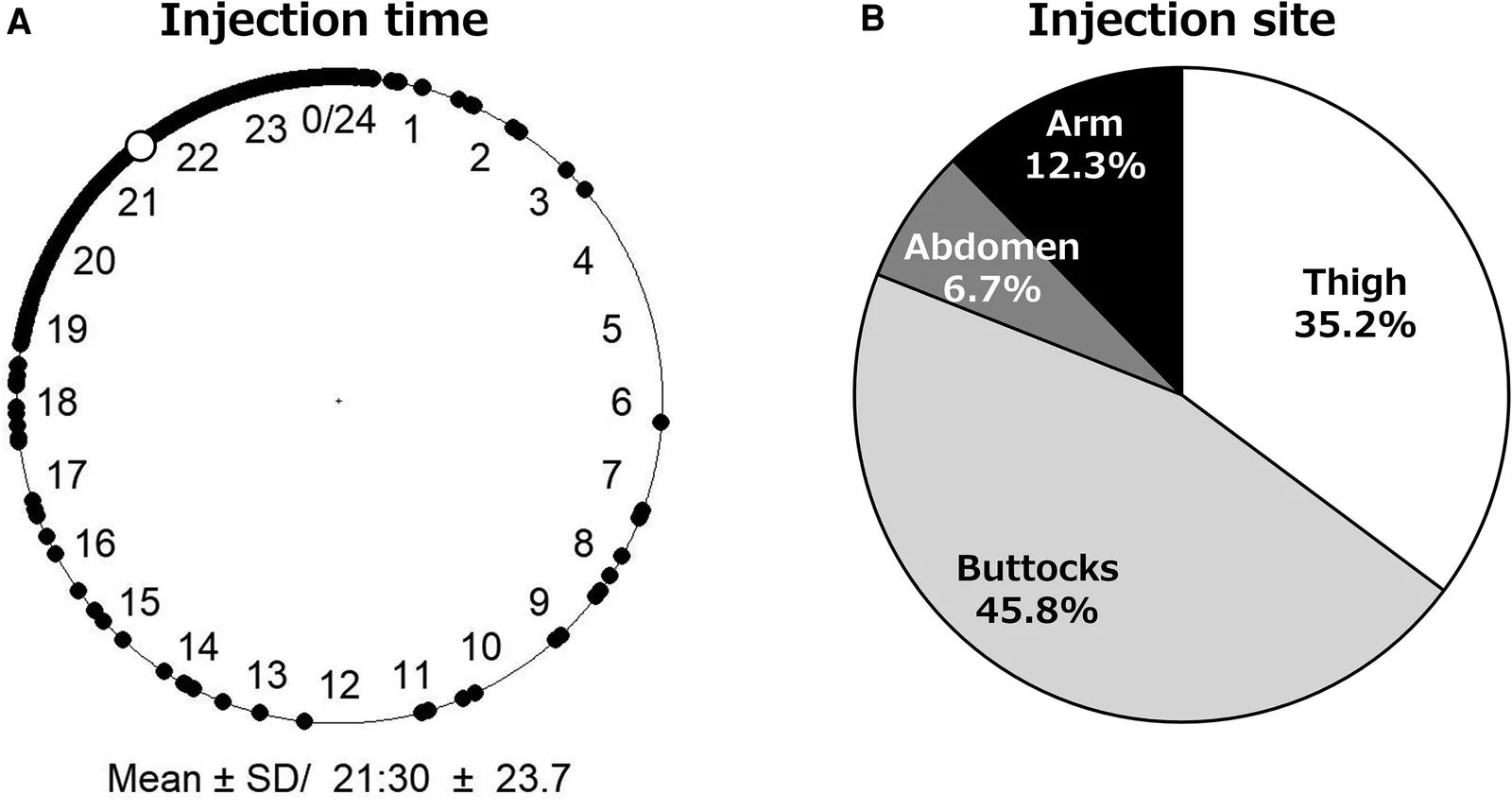
Distribution of (A) mean injection time and (B) injection sites.

We evaluated the timing and anatomical sites of home-based GH injections in the FAS. The mean ± SD injection time was 21:30 ± 23.7 minutes. Injection timing varied widely across patients, with some injections administered between 1:00 and 3:00 or between 6:00 and 9:00 (Fig. 6A). The most frequently used injection sites, in descending order, were the buttocks, thigh, upper arm, and abdomen (Fig. 6B). Age-related differences in injection site preference were observed. Among patients aged 11 years and younger, the buttocks were the most commonly used site, whereas among those aged 12 years and older, the thigh was the most frequently selected site.

### Adherence trends for home-based self-injection of GH and contributing factors

**Figure 7:**
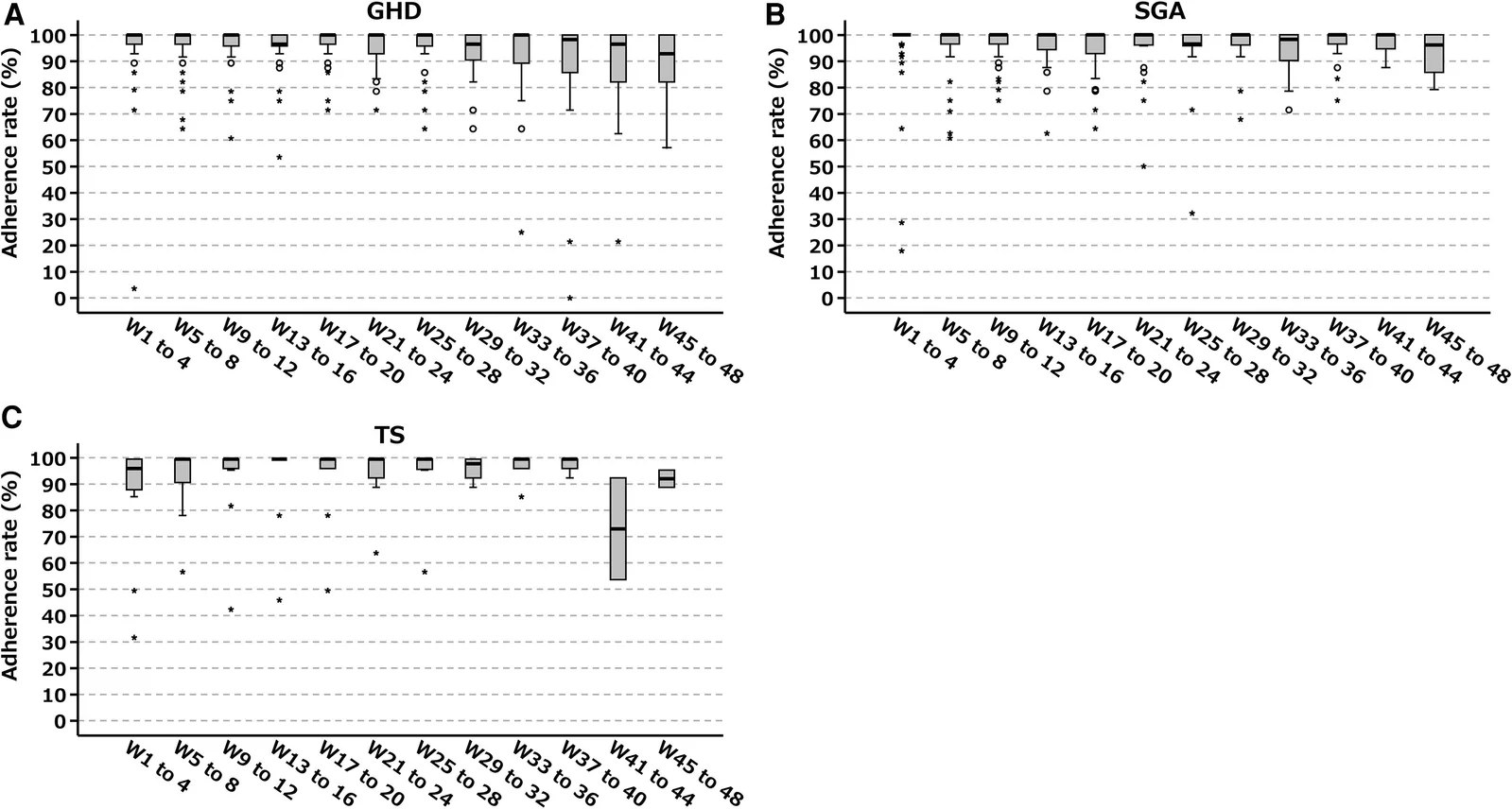
Changes in adherence rates. (A) Patients with GH deficiency. (B) Patients born small for gestational age (SGA). (C) Patients with Turner syndrome and SHOX disorder.

Adherence trends were analyzed by treatment indication in the EDS. In patients with GHD, a decline in adherence was observed after the second month of treatment initiation, followed by a moderate to gradual decrease thereafter (Fig. 7A). Patients with SGA exhibited a moderate or greater decline in adherence beginning after the fourth month (Fig. 7B). Additionally, among patients with SGA, a stratified analysis according to dose increase status at week 24 displayed that adherence rates over the first 6 months of treatment were significantly lower adherence in patients who did not undergo dose escalation compared with those who did. In patients with Turner syndrome or SHOX disorder, the number of cases available for adherence analysis was limited, and no statistically significant differences in adherence trends were identified (Fig. 7C).

**Figure 8:**
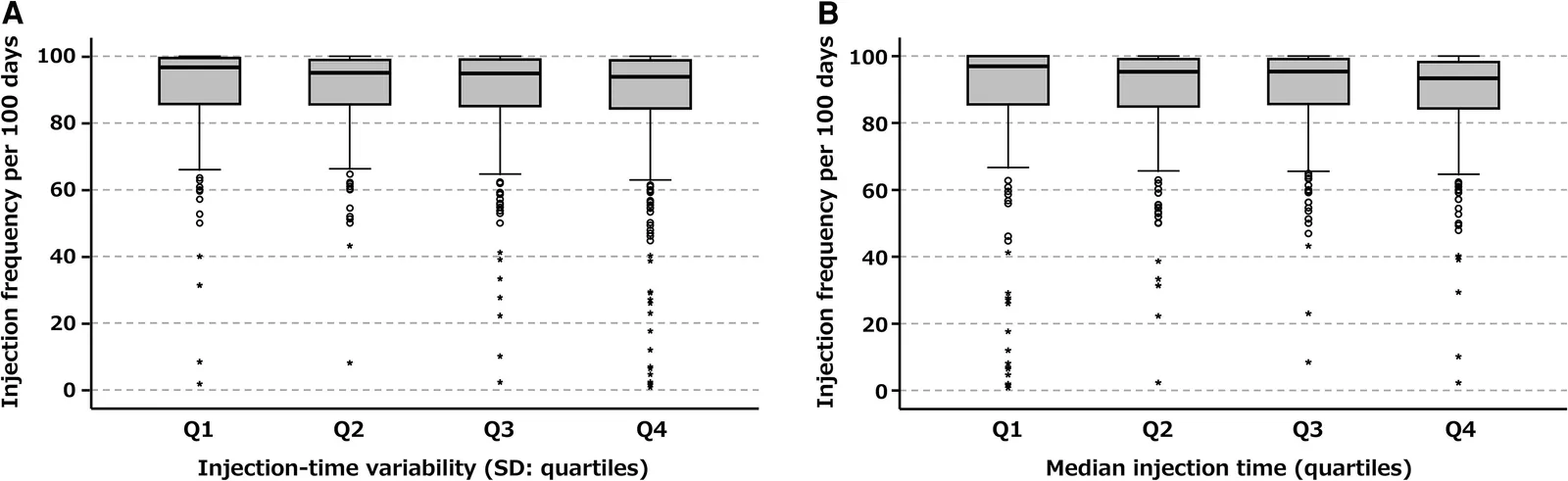
Association between injection frequency per 100 days and (A) variability in injection time (SD quartiles: Q1, ≤9.8 minutes, Q2, 9.9-13.5 minutes, Q3, 13.6-18.8 minutes, Q4, > 18.9 minutes; *P* < .05) (B) injection time quartiles (Q1, −20:45; Q2, 20:45-21:27; Q3, 21:27-22:14, Q4, 22:14–; *P* < .05).

We examined the association between injection time and adherence in the FAS. Generally, daily GH preparations are self-administered via injection once daily, typically after dinner or before bedtime to align with the physiological secretion pattern of GH. Data on home self-injection times showed that adherence rates were lower among those with inconsistent injection times compared to those who injected at the same time daily (Fig. 8A). Adherence was significantly higher among patients with an average self-injection time earlier than 20:45 Pm. and significantly lower among those with an average self-injection time later than 22:14 Pm (Fig. 8B).

### Association between smartphone application usage and injection adherence

**Figure 9:**
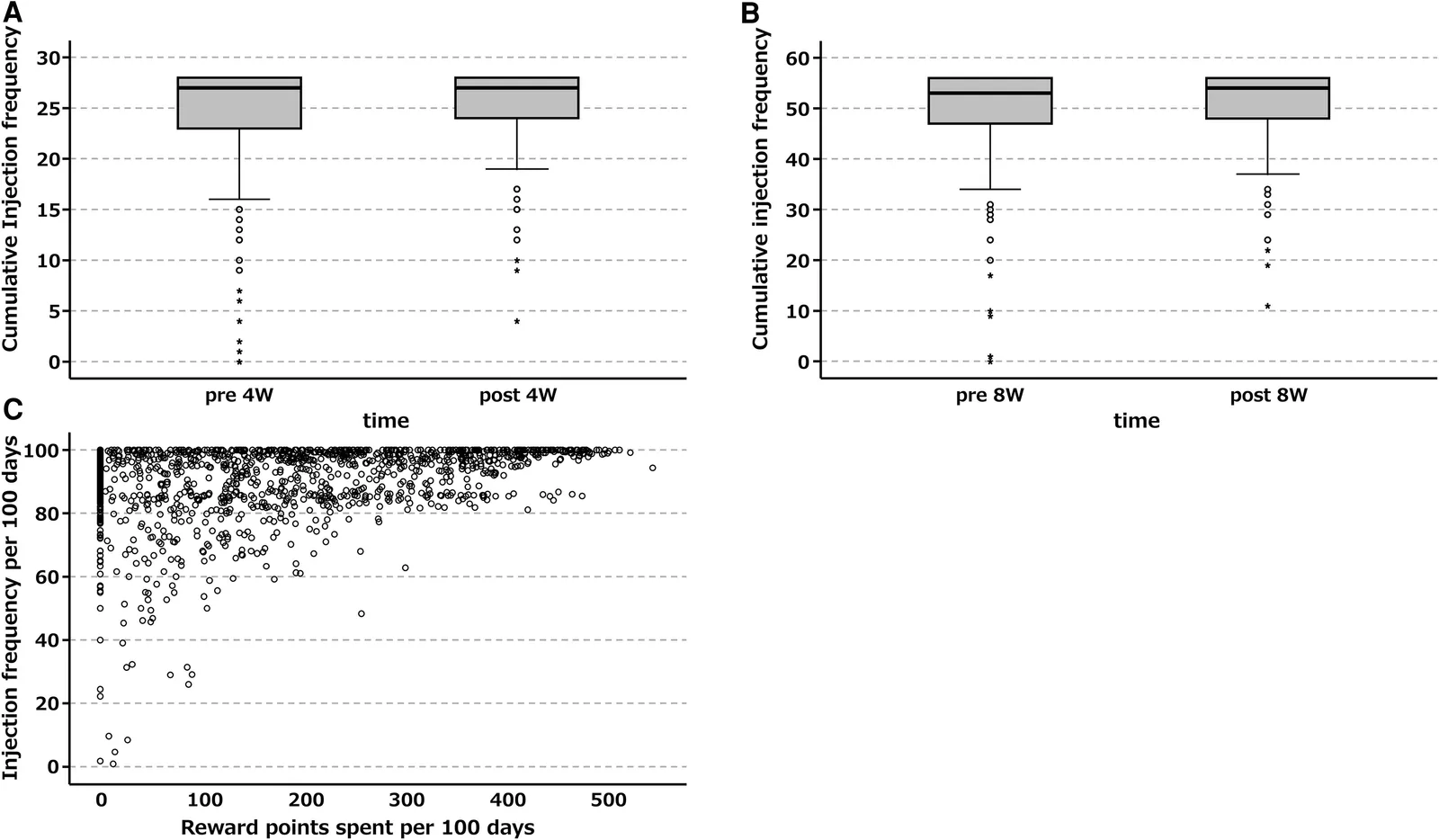
Association between injection frequency and application usage. (A, B) Cumulative injection frequency at 4 and 8 weeks before and after application registration (*P* < .05). (C) Correlation between injection frequency and application points used per 100 days during the treatment period (*P* < .05).

We compared the number of injections administered before and after registration of the smartphone application in the CTS using 4-week and 8-week observation windows. The cumulative number of injections was significantly higher after registration in both analyses (Fig. 9A and 9B). Beyond 8 weeks, the number of injections was comparable between the pre- and postregistration periods. These findings suggest that application registration contributes to adherence during the first 2 months of GH therapy.

Next, we evaluated the association between application usage and injection frequency in the FAS, using point utilization for avatar items as an indicator of engagement. A significant positive correlation was observed between the number of points used per 100 days and the number of injections administered, irrespective of sex (Fig. 9C).

Age-stratified analysis showed a significant association between point usage and adherence among patients younger than 12 years. In contrast, no significant correlation was observed in patients aged 12 years and older. These findings suggest that higher application engagement is associated with improved adherence, primarily among younger patients.

### Multivariate analysis for cumulative adherence rate at 24W

Finally, to identify factors associated with cumulative adherence rate at week 24, we first performed univariate regression analysis, followed by multivariate analysis in the EDS. In univariate analyses, scheduled number of injections per week, variability in injection time, median injection time, and cumulative app usage (as assessed by cumulative point usage up to week 24) were identified as candidate independent variables and were included in the multivariate model. The multivariate analysis showed that cumulative app usage is an independent factor that significantly contributes to an increase in the cumulative adherence rate. Although the wide variation in injection times (SD ≥ 18.9 minutes) did not reach statistical significance, it showed a tendency to reduce the cumulative adherence rate at week 24 (Table 1).

**Table 1:** Univariate and multivariate regression analyses of factors associated with cumulative adherence rate at week 24

Univariate analysis				
Variable	Level	Estimate	Std_Error	*P*-value
Age at the start of observation	(Continuous)	−0.144	0.224	.523
Body height SDS at the start of observation	(Continuous)	−1.328	1.925	.492
Sex ref: male	Female	−1.165	1.636	.479
Indications	TS	−3.85	6.421	.551
Ref: GHD	SGA	0.113	1.393	.936
Number of injection per week ref: 6	7	−1.563	1.278	.**225**
Median injection time	Q2	−0.882	1.951	.653
Ref: Q1	Q3	0.733	1.263	.564
	Q4	−4.274	2.741	.**123**
Injection time SD	Q2	−3.112	2.487	.**215**
Ref: Q1	Q3	−0.213	0.868	.807
	Q4	−4.595	2.109	.**033**
Total points used_W24	(Continuous)	0.008	0.003	.**002**

Multivariate analysis 1				
Term	Level	Estimate	Std_Error	*P*-value
Number of injection per week ref: 6	7	−3.018	2.684	.265
Median injection time	Q2	−2.350	2.920	.424
Ref: Q1	Q3	0.079	1.544	.960
	Q4	−3.663	2.386	.130
Injection time SD	Q2	−3.929	3.240	.230
Ref: Q1	Q3	−1.279	1.644	.439
	Q4	−3.678	2.470	.141
Total points used_W24	(Continuous)	0.007	0.003	**.020**

Multivariate analysis 2: From a clinical perspective, the analysis was conducted excluding cumulative usage points				
Term	Level	Estimate	Std_Error	*P*-value
Number of injection per week ref: 6	7	−2.556	2.565	.323
Median injection time	Q2	−2.004	2.851	.485
Ref: Q1	Q3	0.137	1.512	.928
	Q4	−4.010	2.528	.117
Injection time SD	Q2	−4.112	3.283	.215
Ref: Q1	Q3	−1.228	1.571	.437
	Q4	−4.715	2.646	**.079**

Univariate analyses were performed to screen candidate independent variables for inclusion in the multivariate analysis, using a threshold of *P* < .25. Variables with *P* < .05 in the multivariate analysis were considered independently associated with cumulative adherence rate.

## Discussion

To the best of our knowledge, this study provides the first comprehensive real-world analysis of home-based GH injection practices in Japan using data derived from an application-connected electronic injection device. Our findings elucidate current adherence patterns, growth outcomes, and changes in height and BMI by treatment indication. Regarding adherence by treatment indication, injection adherence declined at approximately 2 months after treatment initiation in patients with GHD and at approximately 4 months in patients with SGA. These time points may therefore represent critical windows for targeted clinical intervention. Our analysis further demonstrated that greater variability in injection timing and delayed injections were associated with lower adherence. These findings may inform practical guidance provided to patients and caregivers. Furthermore, use of the smart application linked to the electronic injection device was significantly associated with improved injection retention during the first 2 months after treatment initiation, suggesting that application-based support may contribute to early adherence maintenance.

Consistent with previous reports, GH therapy was associated with an increase in height SDS of approximately one growth channel, that is the space between each 0.5 SD channels in growth curve, per year [9]. When analyzed by indication, significant height gains were observed across all indications including GHD, SGA, and Turner syndrome or SHOX disorder. Overall, BMI decreased over the first year of treatment, with a more pronounced reduction observed in female patients. Given the established metabolic effects of GH on reducing body fat [9], the observed decrease in BMI is likely attributable to GH therapy. In contrast, BMI changes were not evident in the SGA group over the 1-year treatment period. Given that children with SGA are known to exhibit relative resistance to GH sensitivity [10, 11], it is possible that certain GH-mediated effects, including reductions in body fat, may be attenuated in this population. In our SGA cohort, when height outcomes were analyzed by stratifying patients according to whether a dose increase was implemented at 6 months posttreatment initiation, height SDS at 1 year was comparable between patients with- and without a dose increase. This finding indicates greater GH resistance among patients who required a dose increase.

GH therapy is typically administered at home, and maintenance of adherence is closely associated with treatment efficacy [6, 7]. Traditionally, health care professionals have relied on indirect indicators—such as patient or guardian self-reports, serum insulin growth factor-1 (IGF-1) levels, and growth trajectories—to estimate injection adherence. This reliance has posed a clinical challenge, as delays in accurately assessing adherence can hinder timely situational awareness and appropriate intervention. With recent advances in digital transformation, technologies enabling the collection and analysis of large-scale real-world data have rapidly evolved. In particular, diabetes care has led this shift through the widespread adoption of insulin pumps and continuous glucose monitoring systems that allow real-time access to treatment data [12, 13]. Although digital innovation in GH therapy remains limited, the smartphone application–linked electronic injection device used in this study provides detailed, objective information on injection administration that was previously unavailable.

The study further demonstrated gradual declines in adherence among patients with GHD and those born with SGA, beginning approximately 2 months and 4 months after treatment initiation, respectively. Contrary to initial expectations, daily administration was associated with more stable adherence, suggesting that adherence is more likely to be maintained when GH injections are incorporated into a consistent daily routine. Moreover, adherence was significantly lower among patients who administered injections later in the day or with greater variability in injection timing. These findings indicate that, in home-based therapy, integrating injections into a consistent, regular, and sustainable daily routine may improve adherence. Accordingly, instructing patients and caregivers with poor adherence to maintain consistent injection times and establish durable injection habits may enhance treatment compliance. According to this analysis, some patients were receiving injections late at night. We speculate that some patients were teenager preparing for entrance exams who stayed up late, and that in cases where family members administered the injections, they were often given at late hours. In addition, in patients with SGA, adherence during the first 6 months of treatment was significantly lower in those who did not undergo dose escalation compared with those who did. This finding suggests that appropriate dose adjustment may help support motivation among patients and caregivers to continue treatment. In addition to observable height gains, timely feedback from health care professionals regarding dosage adjustment may be essential for reinforcing adherence and supporting long-term treatment continuation.

In this study, we examined the effectiveness of a smartphone application as a supportive tool for patients and caregivers in maintaining daily injection adherence. Generally, decreased motivation and fatigue have also been known with conventional daily-dose GH formulations during treatment. Previous studies have reported that among the various application features, engagement with entertainment-based content tends to correlate with the continued use of growth-tracking functions [8]. Consistent with these findings, patients in our cohort with higher usage point utilization demonstrated significantly higher adherence rates. Furthermore, during the first 2 months before and after application registration, adherence was significantly higher after registration compared to before registrations. These findings demonstrate that smart applications linked to electronic injection devices, particularly when combined with reward points for injection, can effectively enhance adherence to GH injections. Notably, this effect was observed primarily in participants aged 11 years old and younger. For patients aged 12 years and older, the development of applications incorporating alternative motivational strategies remains a challenge.

Previous studies investigating treatment adherence in children aged 8 to 18 years with type 1 diabetes have shown that impulse control and self-efficacy positively influence adherence behaviors. Self-efficacy refers to an individual's perceived confidence in their ability to perform self-care behaviors under difficult or emotionally challenging conditions [14]. In the present study, the electronic injection device incorporated a reward-based system in which points were accrued with each injection, allowing users to redeem accumulated points for preferred avatar items. Each administered injection was accompanied by tangible incentives, including point accumulation, achievement of injection milestones, and intrinsic motivation derived from personalized rewards. In addition to daily injections, gaining the confidence and self-efficacy from the experience of achieving the goal of increased height may further reinforce treatment engagement among pediatric patients.

This study has several limitations. First, the analysis was restricted to the first year after treatment initiation, and the number of cases for certain indications was insufficient for robust subgroup analyses. Second, as a retrospective study, potential selection bias related to age and sex may exist, as participation was limited to patients who chose to use this electronic injection device. Third, since this study relied on an analysis of usage data stored in the electronic device, we were unable to obtain blood data such as patients’ IGF-1 levels.

Despite these limitations, this study demonstrated that injection adherence begins to decline approximately 2 to 4 months after treatment initiation, suggesting that clinical intervention may be necessary at or before this time point. Use of the smartphone application during the initiation phase was shown to support proactive and sustained adherence. Future challenges include developing age-appropriate application features and improving real-time sharing of treatment data with health care professionals, which may further enhance long-term adherence.

## Data Availability

Some or all datasets generated during and/or analyzed during the current study are not publicly available but are available from the corresponding author on reasonable request.
