# Correction to: “Adherence to Daily Growth Hormone Therapy in Japan: Real-World Data from the Melon Nikki™ App and Device”

**DOI:** 10.1210/jendso/bvag213

**Published:** 2026-09-24

In the above-named article by Nakamura-Utsunomiya A, Ono T, and Arashin O (J Endo Society. 2026; 10(6); doi: 10.1210/jendso/bvag113), there was an error in the abstract.

In the originally published article, in the Context section of the abstract, a sentence reads, “Since 2020, growth hormone therapy administered using electronic drug delivery devices linked to a smartphone application has been approved in Japan.”

In the corrected article, the sentence has been corrected to “Since 2020, a smartphone app (Melon Nikki^TM^) that is used in conjunction with the electronic drug delivery device (GROWJECTOR^TM^L) has been available in Japan.”

The article has been corrected online.

doi: 10.1210/jendso/bvag113

